# Risk factors and relationship between screening periodicity and risk of cervical cancer among nurses and midwives. A cross-sectional study

**DOI:** 10.1590/1516-3180.2018.0244230119

**Published:** 2019-07-15

**Authors:** Selda Yörük, Ayla Açıkgöz, Hülya Türkmen, Gül Ergör

**Affiliations:** I PhD. Assistant Professor, Department of Midwifery, School of Health, Balıkesir University, Cagis Campus, Balıkesir, Turkey.; II PhD. Lecturer, Vocational School of Health Services, Dokuz Eylül University , Izmir, Turkey.; III PhD. Midwifery Lecturer, Department of Midwifery, School of Health, Balıkesir University, Cagis Campus, Balıkesir, Turkey.; IV MD. Professor, Department of Public Health, Faculty of Medicine, Dokuz Eylül University Izmir, Turkey.

**Keywords:** Nurse midwives, Uterine cervical neoplasms, Papanicolaou test

## Abstract

**BACKGROUND:**

If nurses and midwives undergo cervical cancer screening regularly, they can become role models for other women regarding this screening.

**OBJECTIVES:**

The aims here were (i) to determine factors associated with undergoing cervical cancer screening; and (ii) to examine the association of cervical cancer screening periodicity with cervical cancer risk levels among nurses and midwives.

**DESIGN AND SETTING:**

Cross-sectional study in a public hospital.

**METHODS:**

466 nurses and midwives participated in this study. The relationships between undergoing Pap smear screening and sociodemographic characteristics, cervical cancer risk factors, perception of cervical cancer risk and calculated cervical cancer risk levels were examined. Cervical cancer risk levels were determined using the “Your Disease Risk” assessment tool (Washington University).

**RESULTS:**

35% of the nurses and midwives had undergone Pap smear testing at least once in their lifetimes. The odds of having undergone Pap smear testing were higher among smokers (odds ratio, OR: 2.08; 95% confidence interval, CI: 1.24-3.48) and among those who perceived their risk of cervical cancer to be high (OR: 3.60; 95% CI: 1.36-9.51). The frequency of undergoing Pap smear testing at least once in a lifetime was higher among primiparae (OR: 17.99; 95% CI: 6.36-50.84) and secundiparae (OR: 41.53; 95% CI: 15.01-114.91) than among nulliparae. No relationship was found between Pap smear test periodicity and calculated risk level.

**CONCLUSION:**

There is a need to assess motivational barriers that might lead to low levels of Pap smear screening among nurses and midwives who are role models for women regarding cervical cancer prevention.

## INTRODUCTION

Cervical cancer screening using Pap smear tests is a cost-effective method for preventing cancer.^1^^,^^2^ Diagnosing and treating cervical cancers at the premalignant stage will decrease both incidence and mortality.^1^ In the United States, Finland, Sweden, Denmark and Norway, the incidence of cervical cancer has been significantly reduced through the use of Pap smear test screening.^3^^,^^4^

However, in developing countries such as Turkey, the incidence of cancer is gradually increasing.^5^ This increase reveals that there is a need for effective application of cancer screening programs. The American Cancer Society has recommended that all women should start to undergo cervical cancer screening from the age of 21, and that women aged 30-65 years should undergo Pap smear screening once every three years.^6^ In Turkey, Pap smear test screening is recommended for women aged 30-65 years within the scope of the National Cancer Control Program.^7^ If a person has a high risk of cervical cancer, it is recommended that testing should be done from an early age.^4^

Nurses and midwives work at every stage of the healthcare system and are the professionals who come into direct contact with society. Nurses and midwives’ lifestyles relating to cervical cancer and the degree to which they self-apply methods for early diagnosing of this condition are indicative of their individual awareness within this field. Positive attitudes and behavior regarding Pap smear testing among healthcare professionals may contribute towards prevention of cervical cancer both for themselves and for the community.^8^

Despite the role of nurses and midwives in cervical cancer screening, there is little knowledge about Turkish nurses and midwives’ own cervical cancer screening behavior. Studies conducted among healthcare professionals in Turkey have focused more on the frequency of screening.^9^^,^^10^^,^^11^^,^^12^^,^^13^ Only two of these studies examined the factors affecting whether individuals choose to undergo Pap smear tests. It was determined that age affected screening behavior,^9^ and that having information on risk factors did not affect screening behavior.^12^ However, we did not find any studies in which cervical cancer risk levels among midwives and nurses were examined.

## OBJECTIVES

The aims of this study were (i) to determine factors associated with undergoing cervical cancer screening; and (ii) to examine the association of cervical cancer screening periodicity with cervical cancer risk levels among nurses and midwives working in two Turkish public hospitals.

## METHODS

The potential participants for this cross-sectional study comprised female nurses and midwives working in two different hospitals located in the province of Balıkesir in Turkey (n = 680). These hospitals are the largest public hospitals in this province, and the highest numbers of nurses and midwives work in these institutions. No sample selection was conducted for this study: the aim was to reach the entire potential population.

Prior to undertaking the study, written permissions were obtained from the Balıkesir University Ethics Committee for Non-Interventional Studies and from the managements of the two hospitals. Participants gave their informed consent to be included in this study.

Research data were collected between January 2016 and September 2017. The data were collected through a survey that was created by the present researchers taking into consideration information from the literature within this field. The survey questionnaires were distributed to all midwives and nurses at the institutions where they worked, in closed envelopes. The questionnaire consisted of questions to determine the survey participants’ educational background, marital status, cervical cancer risk perception and frequency of undergoing Pap smear tests. It also included questions taken from the “Your Disease Risk” program,^14^ which were used to calculate the level of cervical cancer risk.

There were 202 nurses and midwives who refused to participate in the study. A further 12 midwives and nurses were excluded from the study because they did not complete the survey. Thus, a total of 466 nurses and midwives fully filled out the questionnaire. The response rate was therefore 68.5%.

In order to determine the cervical cancer risk level, the risk assessment tool “Your Disease Risk”, published by the Washington University School of Medicine, was used.^14^ In the risk calculation software, assessments were made according to the following features: age; histories of cancer, human immunodeficiency virus (HIV), hysterectomy, smoking and sexually transmitted diseases (STDs); number of sexual partners; age at the time of initial sexual intercourse; use of condoms; number of children delivered; and whether any Pap smear tests had been undertaken over the last three to five years. The questions asked through this software were added to the questionnaire.

In the risk calculation model for cervical cancer, there were seven risk categories: very much below average, much below average, below average, average, above average, much above average and very much above average. In our study, the cervical cancer risk among the participants was found to be “average”, “below average” or “much below average”. These three groups were analyzed in the chi-square test. In examining the relationship between risk levels and experience of having undergone Pap smear tests, the cervical cancer risk levels “below average” and “much below average” were combined into a single group, named the “low” group, and in the logistic regression analysis, the assessment was thus made in terms of two groups, named the “average” and “low” groups.

For analytical purposes in the chi-square test, the following variables were dichotomized: marital status (currently married versus unmarried); smoking status (currently smoking versus non-smoker); and having given birth (having given birth versus not having given birth). The following variables were analyzed as three groups: age (20-29 years versus 30-39 versus ≥ 40); cervical cancer risk perception (high versus low versus unknown); and calculated cervical cancer risk level (average versus below average versus much below average). In the logistic regression analysis, age (≥ 30 years versus < 30) and calculated cervical cancer risk level (average versus low) were dichotomized. The number of births was divided into four groups (no births, one birth, two births or three births). The frequency of having Pap smear screening was assessed in four groups (first time, once a year, once every 2-3 years or at irregular intervals) and the latest Pap smear screening was assessed in four groups (within one year, 1-3 years ago, 4-5 years ago or more than five years ago).

The data were analyzed through the Statistical Package for the Social Sciences (SPSS) for Windows 20.0 statistical software. Continuous variables were presented as means and standard deviations; and categorical variables were presented as numbers and percentage distributions. The characteristics of participants who had undergone Pap smear testing once in their lifetimes and those who had not been screened were compared using the chi-square test for categorical variables and using Fisher’s exact test for categorical variables with small cell counts. To investigate factors associated with screening for cervical cancer, we used simple and adjusted logistic regression models. The variables that were found to be statistically significant through the chi-square test were included in the logistic regression model. The statistical significance level was taken to be P < 0.05.

## RESULTS

The mean age of the participants was found to be 33.3 ± 7.4 (minimum = 22; maximum = 55). 35.0% of the participants had undergone Pap smear testing at least once in their lifetimes. The most common reason for not having undergone Pap smear testing was found to be negligence (Table 1).

**Table 1.** Proportions of nurses and midwives who had and had not undergone Pap smear testing and reasons for not undergoing any tests

	Number	%
Pap smear test		
Done	163	35.0
Not done	303	65.0
Reasons for not having undergone Pap smear testing		
Not knowing that it is necessary	14	4.6
Neglect	199	65.7
Afraid of undergoing a smear test	17	5.6
Ashamed of undergoing a smear test	13	4.3
Afraid of cervical cancer	2	0.7
Being single	58	19.1

The characteristics of the participants according to screening status are shown in Table 2. The incidence of having undergone Pap smear testing at least once in a lifetime was higher among subjects who were older, who were smokers, who had given birth, who had histories of STD, who had histories of chronic diseases, who perceived their risk to be high and who had average risk of cervical cancer **(**Table 2**)**. These significant variables were examined together in the logistic regression model.

**Table 2.** Comparison of characteristics between participants who had been screened once in their lifetimes and participants who had not (n = 466)

Characteristics	Pap smear test				P
	Done		Not done		
	Number (n = 163)	%	Number (n = 303)	%	
Age group (years)					
20-29	45	27.6	123	40.6	0.001
30-39	64	39.3	152	50.2	
≥ 40	54	33.1	28	9.2	
Educational status					
High school	25	15.3	51	16.8	0.326
Associate’s degree*	64	39.3	98	32.3	
Bachelor’s degree	74	45.4	154	50.9	
Marital status					
Married	140	85.9	183	60.4	0.001
Unmarried	23	14.1	120	39.6	
Smoking status					
Smoker	65	39.9	67	22.1	0.001
Non-smoker	98	60.1	236	77.9	
Childbirth					
Having given birth	155	95.1	139	45.9	0.001
Not having given birth	8	4.9	164	54.1	
Live birth count					
1 birth	38	24.5	59	42.2	0.004
2 births	99	63.9	70	50.4	
3 births	18	11.6	10	7.2	
Regular use of condom in sexual intercourse					
User	19	11.7	27	8.9	0.343
Non-user	144	88.3	276	91.1	
History of STDs					
Present	7	4.3	1	0.3	0.003
None	156	95.7	302	99.7	
History of chronic diseases					
Present	24	14.7	23	7.6	0.015
None	139	85.3	280	92.4	
Response to the question: “Do you think you have a high risk of cervical cancer?”					
“Yes”	38	23.3	25	8.3	0.001
“No”	12	7.4	40	13.2	
“Don’t know”	113	69.3	238	78.5	
Calculated cervical cancer risk					
Average	16	9.8	12	4.0	0.001
Below average	49	30.1	60	19.8	
Much below average	98	60.1	231	76.2	

STD = sexually transmitted disease.

*Associate’s degree programs take two years. High school graduates can qualify for associate’s degree programs.

The incidence of having undergone Pap smear testing was higher among smokers than among non-smokers (odds ratio, OR: 2.08; 95% confidence interval, CI: 1.24-3.48) and among those who stated that they perceived their risk of cervical cancer to be high, compared with those who perceived their risk to be low (OR: 3.60; 95% CI: 1.36-9.51). The frequency of having undergone Pap smear testing at least once in a lifetime was higher among participants who had given birth once (OR: 17.99, 95% CI: 6.36-50.84) and among participants who had given birth twice (OR: 41.53, 95% CI: 15.01-114.91) than among those who had never given birth (Table 3).

**Table 3.** Factors associated with screening for cervical cancer among midwives and nurses (n = 466)*

Variables	Unadjusted OR (95% CI)	Adjusted OR (95% CI)	P
Age group			0.753
Age ≥ 30	1.79†† (1.18-2.72)	1.08 (0.65-1.79)	
Age < 30**	1.00	1.00	
Marital status			0.170
Married	3.98†† (2.44-6.65)	0.54 (0.22-1.29)	
Unmarried**	1.00	1.00	
Smoking status			0.005
Smoker	2.33†† (1.53-3.53)	2.08 (1.24-3.48)	
Non-smoker**	1.00	1.00	
Response to the question: “Do you think you have a high risk of cervical cancer?”			
Yes	4.99†† (2.22-11.68)	3.60 (1.36-9.51)	0.010
Don’t know	1.58 (0.81-3.24)	1.40 (0.62-3.10)	0.414
No**	1.00	1.00	
Cervical cancer risk level			
Average	2.63†† (1.20-5.85)	1.00 (0.64-0.74)	0.999
Low**	1.00	1.00	
Number of births			
1 birth	13.06 (5.93-31.47)	17.99 (6.36-50.84)	0.001
2 births	28.67 (13.75-66.34)	41.53 (15.01-114.91)	0.001
3 births	35.37 (12.68-107.02)	1.40 (0.23-0.41)	0.900
Not having given birth**	1.00	1.00	

*A model was created based on age, marital status, number of births, smoking status, risk perception and calculated cervical cancer risk level; **Reference value.

OR = odds ratio; CI = confidence interval.

Ninety-four percent of the participants were found to have a low level of cervical cancer risk. No relationships were detected between Pap smear test periodicity and the calculated risk level (Table 4).

**Table 4.** Comparison of incidence of Pap smear testing according to cervical cancer risk level (n = 163)

	Cervical cancer risk level				P*
	Average		Low		
	Number (n = 16)	%	Number (n = 147)	%	
Frequency of undergoing Pap smear testing					
First time	5	31.3	34	23.1	0.534
Once a year	1	6.2	27	18.4	
Once every 2-3 years	4	25.0	30	20.4	
Irregular intervals	6	37.5	56	38.1	
Latest Pap smear screening					
Within last year	7	43.8	67	45.6	0.946
1-3 years ago	4	25.0	42	28.6	
4-5 years ago	3	18.8	13	8.8	
More than 5 years ago	2	12.5	25	17.0	

*Fisher exact test.

## DISCUSSION

This study determined the association between having undergone Pap smear testing and cervical cancer risk levels among female nurses and midwives who were providing services in two hospitals. In this study, about one-third of the participants had undergone Pap smear testing at least once in their lifetimes. The most common reason for not having undergone Pap smear testing was found to be negligence. The incidence of having undergone Pap smear testing once in a lifetime was higher among smokers. This rate was higher among the individuals who stated that they perceived their risk of cervical cancer to be high, compared with those who perceived their risk to be low. The frequency of having undergone Pap smear testing at least once in a lifetime was higher among those who had given birth once or twice, compared with those who had never given birth. The majority of the participants had low risk levels. No relationships were found between Pap smear test periodicity and the calculated risk level.

Thirty-five percent of the nurses and midwives who participated in this study had undergone Pap smear testing at least once in their lifetimes. The frequency of having undergone Pap smear testing at least once in a lifetime was found to range from 10.2% to 83.7% in studies conducted in Turkey and Serbia among women.^15^^,^^16^ This rate was found to range from 23.7% to 45.2% among healthcare professionals in Turkey.^9^^,^^10^^,^^11^^,^^12^^,^^13^ In studies conducted among nurses in different countries, the incidence of Pap smear testing at least once in their lifetimes ranged from 3.4% to 72.6%.^17^^,^^18^^,^^19^^,^^20^^,^^21^^,^^22^ The frequency of having undergone Pap smear testing was higher in our study than in some studies^17^^,^^18^^,^^19^^,^^20^ and lower than in other studies.^21^^,^^22^ In addition to factors such as age, education and presence of a screening program in a given country, it has been shown that the level of development of countries also has an effect in relation to cervical cancer screening.^8^ Studies in which the incidence of Pap smear testing was lower than in our study were conducted in underdeveloped or developing countries.^17^^,^^18^^,^^19^^,^^20^

About two-thirds of the nurses and midwives who participated in this study stated that they had never undergone Pap smear testing. In this study, it was determined that for most of the nurses and midwives who had not undergone Pap smear testing, this was due to their own negligence (65.7%), according to their responses to the questionnaire. In other studies among nurses and midwives, the most common reasons that they stated for not having undergone such tests were factors such as not considering themselves to be at risk,^18^^,^^19^ thinking that screening was not necessary for themselves^11^^,^^23^ and negligence.^13^ Encouragement by the partner, regular examination by a gynecologist and suggesting a Pap smear test by these experts are effective measures in increasing the regular undertaking of the Pap smear test.^22^^,^^24^ In-service training can be given to nurses and midwives regarding the importance of cervical cancer risk factors and of undergoing Pap smear tests.

In the present study, it was found that the incidence of undergoing Pap smear testing was higher among smokers. Higher levels of carcinogenic substances were found in the cervical mucus of smokers than in the mucus of non-smokers. It is thought that these substances may be effective in aiding development of cervical cancer through causing damage to the deoxyribonucleic acid (DNA) of cervical cells.^4^ In some studies conducted among healthcare professionals, the levels of information regarding the fact that smoking is a risk factor for cervical cancer were found to be low.^11^^,^^13^ On the other hand, in other studies, although these levels were found to be high, only low levels of screening were occurring.^12^^,^^23^ In our study, the odds of undergoing Pap smear testing were higher among smokers. This significant finding may have been caused by the fact that because of their profession, the research group of this study knew about the fact that smoking is an important risk factor for cervical cancer.

In the study group, the odds of having undergone Pap smear testing were higher among participants who perceived themselves to be at risk in terms of cervical cancer. Women’s perceptions of risk relating to cervical cancer are an important subjective finding: this perception leads them to maintain a healthy lifestyle and take advantage of early diagnostic methods. Perception of high risk of cervical cancer leads women to increase their frequency of undergoing screening.^25^ 13.5% of the nurses and midwives who participated in the present study perceived their own risk of cervical cancer as high. In a study conducted among nurses working in a hospital in Turkey, 31.8% of them indicated that they perceived themselves to be in the at-risk group in terms of cervical cancer.^13^ The proportion of nurses in a study conducted in Singapore who perceived that they had a high level of individual cervical cancer risk was lower than what was found in the present study.^22^ In a study conducted in a group undergoing treatment for high-grade cervical intraepithelial neoplasia, it was found that 64% of these women perceived themselves to be at high risk and 30% at low risk.^26^

Multiparity has been linked with higher risk of cervical cancer in women.^2^ In the present study, the frequency of having undergone Pap smear testing at least once in a lifetime was higher among participants who had given birth once or twice than among those who had never given birth. In a study conducted among nurses in India, it was also found that multiparity increased the frequency of Pap smear testing.^27^ However, no relationship was found between the frequency of having undergone Pap smear testing and the number of births, in studies conducted in the general population in India.^25^^,^^28^ In the present study, in univariate analysis (chi-square test), it was determined that the incidence of having undergone Pap smear testing increased significantly with increasing age among the nurses and midwives. However, this relationship was not observed in the logistic regression analysis. Among nurses, the incidence of having undergone Pap smear testing was found to be highest in the 40 to 44-year age group in Australia,^21^ in the 35 to 49-year age group in Singapore^22^ and in the group aged 40 years and over in Turkey.^9^

It is quite difficult to calculate the risk of cervical cancer in absolute terms. However, calculating the individual risk of cervical cancer using the risk calculation models that have been developed may provide a guide in terms of both cervical cancer prevention and early diagnosis.^29^ Nevertheless, it is important to accurately compile risk factors from women in determining the risks. In the present study, the calculated level of cervical cancer risk was low in 94% of the nurses and midwives, while only 6% of them had an average level of risk. In a study conducted among women aged 35-69 years, it was calculated that 8% of them had a high level of cervical cancer risk, while 22% of them had an average level of risk and 70% of them had a low level of risk.^15^ In that study, the higher level of risk found among the participants may have resulted from the older age of the participants in that study, compared with those of the present study, along with the higher level of risky behavior among the participants in that study. The low risk presented by the majority of the present study group was due to its young age, with relatively few risky behavioral characteristics. The number of women undergoing treatment for STDs was minimal in the present study.

The risky behavior among the participants that needed to be taken into account in calculating the cervical cancer risks originated from smoking and failure to undergo Pap smear testing over the past three years. About a third of the women in the present study continued to smoke. Açıkgöz et al. found that the incidence of undergoing Pap smear testing decreased as the level of cervical cancer risk increased.^15^ However, in the present study, in the univariate analysis (chi-square test), as the level of cervical cancer risk increased, the incidence of having undergone Pap smear testing increased significantly. On the other hand, this relationship was not observed in the logistic regression analysis. Regardless of which part of society women belong to or at which age they are, it needs to be ensured that they can undergo regular Pap smear testing and that their risky behavior is reduced.

The strengths of the present study include the fact that, although several knowledge-attitude-behavior studies on cervical cancer among nurses and midwives in Turkey were found to be available through our review of the literature, there were none in which the level of cervical cancer risk was determined among nurses and midwives. For the first time in Turkey, nurses and midwives’ cervical cancer risk levels were identified using risk level determination software.

The limitations of our study relate mainly to data collection. We collected self-reported data. Although self-reporting is an acceptable method of data collection in public health studies, it is associated with the potential for recall bias. Therefore, the results regarding the women’s risk levels may have been determined in different manners. The results from this study cannot be generalized to all nurses and midwives. Since the participants did not respond to any question regarding how many partners they had had, the cervical cancer risk level among these individuals may have been lower than the true level because they were considered to be monogamous.

It is undeniable that healthcare professionals have a role to play in raising awareness about cervical cancer within the community. It is necessary to conduct studies aimed at ascertaining what the motivational barriers are that lead to such low levels of Pap smear test screening among nurses and midwives who are role models for women regarding prevention of cervical cancer. Correct information about cervical cancer risk factors and the importance of Pap smear test screening is needed not only for women in the at-risk group but also for nurses and midwives. In this way, socially more effective results regarding prevention of cancer and use of early diagnostic and treatment methods will be obtained.

## CONCLUSION

It was found that the incidence of having undergone Pap smear testing at least once in a lifetime among nurses and midwives was low in this study. Smokers, participants with a high perception of cervical cancer risk and those who had given birth presented higher levels of having undergone Pap smear testing at least once. Most of the nurses and midwives presented low cervical cancer risk levels. No relationships were found between Pap smear test periodicity and the calculated cervical cancer risk level.
